# King Edward's Hospital Fund for London

**Published:** 1908-03-28

**Authors:** 


### HOSPITAL ADMINISTRATION.

### CONSTRUCTION AND ECONOMICS.

### KING EDWARD'S HOSPITAL FUND FOR LONDON.

The annual meeting of the President and General Council of King Edward's Hospital Fund for London, to receive the accounts and the report of the General Council for the year 1907, was held at Marlborough House on the 20th inst., His Royal Highness the Prince of Wales, President of the Fund, being in the chair.

Lord Rothschild (the honorary treasurer) presented the account of receipts and expenditure and the balance sheet for the year ending December 31, 1907. The Prince of Wales moved the adoption of the accounts, which was seconded by Lord Strathcona, and carried unanimously. Sir Savile Crossley (honorary secretary) read the draft report of the Council for the year 1907.

The Prince of Wales, in moving the adoption of the report, said: My Lords and Gentlemen,—The draft report which has just been read shows that the Fund has had a busy year. With reference to the accounts you will be gratified to know that the expenses necessarily incidental to the passage of our Act of Parliament have, by the kindness and generosity of all who have acted for us in the matter, proved comparatively light. Since our Council meeting in December, acting on the advice of the Executive Committee, and in accordance with the powers given under the Act, I have decided to appoint a committee to inquire into the system prevailing in the London voluntary hospitals regarding the admission of out-patients. It is to be hoped that the labour involved in the holding of such an inquiry may perhaps, to some extent, be lightened by the results of the work of the Royal Commission on the Poor Laws, the issue of whose report we look forward to with great interest. In so far as the Commission may deal with certain aspects of the question of medical relief, we cannot fail to profit by a perusal of the evidence given before them, and of the conclusions at which they arrive. We shall, therefore, defer our inquiry until the Commission has reported. Another matter to be brought to your notice is a development of the scope of the operations of the Fund, which I have approved upon the recommendation of the Distribution Committee. Hitherto the hospitals with which we have dealt have been those lying in the County of London, or within seven miles of Charing Cross. This limitation was wisely imposed when the Fund was instituted in 1897. But in the eleven years which have since elapsed London has grown rapidly, and it is felt that the time has come when we should enlarge our area. The radius from Charing Cross has, therefore, been extended from seven to nine miles. We also propose this year to consider the claims of consumption sanatoria in the country, which take a large proportion of their patients from London. It must be obvious that such institutions are better situated in the country than in London or even in the suburbs. These extensions of our work will not, at any rate this year, involve very great additional demands upon the Fund's resources. But a larger area may necessitate a larger distribution, and we must not forget that, even for the smaller area, there has been an ever steadily increasing distribution. The total of last year's distribution exceeded the income from investments by £70,000. With the extended area an increased income from subscriptions and donations will be necessary, and I trust that the public will recognise this and continue to give us their substantial support. The re-arrangement of the work of the Fund necessitated by the Act has undoubtedly added largely to the responsibilities of the honorary secretaries, and increased their work, and I fear that circumstances have recently thrown much of this upon Mr. Fry. To him especially, and to all those who have given their services, I wish to tender my most sincere thanks. I now move the adoption of the report.

The resolution was seconded by the Lord Mayor (Sir John Bell), supported by the Chairman of the London County Council (Mr. R. A. Robinson) and by Lord Bessborough, Chairman of the Executive Committee, and carried unanimously. The Bishop of Southwark moved, and Lord Revelstoke seconded, a vote of thanks to the Prince of Wales for presiding, which was carried by acclamation.

His Royal Highness said: My Lords and Gentlemen,—I thank you for your kind vote of thanks which you have so cordially received. I am indeed gratified at the present most satisfactory state of the Fund. These excellent results are due to the many good friends who support me and who carry out the hard work—and it is very hard work—of administering this large sum of money. It is impossible for me to express sufficiently my gratitude to them for all they do in helping the Fund.

